# Age, emotional burden and deep brain stimulation electrode location shape Parkinson’s disease quality of life

**DOI:** 10.1038/s41746-026-02828-7

**Published:** 2026-06-05

**Authors:** Shiva Khoshnoud, Farzin Negahbani, Idil Cebi, Daniel Weiss, Alireza Gharabaghi

**Affiliations:** 1 Institute for Neuromodulation and Neurotechnology, University Hospital Tübingen, Faculty of Medicine, University Tübingen, Tübingen, Germany; 2 Center for Neurology, Department for Neurodegenerative Diseases, and Hertie Institute for Clinical Brain Research, University Tübingen, Tübingen, Germany; 3 Center for Digital Health (CDH), Tübingen, Germany; 4 Cognitive Science Center (CSC), Tübingen, Germany; 5 Center for Bionic Intelligence Tübingen Stuttgart (BITS), Tübingen, Germany; 6 German Center for Mental Health (DZPG), Tübingen, Germany; 7 Max Planck-University of Toronto Centre for Neural Science & Technology (MPUTC), Toronto, ON Canada

**Keywords:** Medical research, Neurology, Neuroscience

## Abstract

Postoperative quality-of-life (QoL) outcomes after subthalamic deep brain stimulation in Parkinson’s disease vary widely. Previous studies based on PDQ-39 summary scores have reported opposing relationships between baseline and postoperative QoL, reflecting analytic variability, measurement noise, and limited feature scope. To address these inconsistencies, we analyzed 130 patients using an explainable random-forest classifier with SHAP analysis trained to predict QoL changes exceeding minimal clinically important difference thresholds. Baseline variables included PDQ-39 subscores, along with demographic, motor, cognitive, and affective measures, and electrode coordinates derived from imaging. Predictors of QoL improvement included younger age, greater preoperative disease-related emotional burden, and electrode placement at the motor-associative transition in the right subthalamic nucleus. The model achieved an area under the curve of 0.70 on the held-out test set, with balanced sensitivity and specificity. Identifying interpretable cut-offs for age, emotional burden and electrode location supports individualized counseling and treatment planning, advancing outcome prediction in neuromodulation.

## Introduction

Enhancing health-related quality of life (QoL) is a central goal in the management of Parkinson’s disease (PD), where QoL is often markedly reduced^1^. Unlike clinician-rated motor evaluations, QoL metrics such as the PDQ-39 capture the broader physical, emotional, and social burden of PD; however, identifying consistent predictors of QoL change has proven difficult, because QoL reflects the complex interplay of motor and non-motor symptoms, psychological state, and disease progression^2^.

Deep brain stimulation (DBS) of the subthalamic nucleus (STN) effectively alleviates motor symptoms in advanced PD, yet its impact on QoL remains variable^3^. Although many patients experience meaningful improvement, others show minimal benefit or even decline^4–8^. Studies investigating predictors of QoL outcomes have reported conflicting results^9^. Some have linked greater preoperative QoL burden to stronger postoperative gains^6,10–12^, whereas others found the opposite relationship^13,14^.

A closer review of prior work indicates that these apparent contradictions may arise at least in part from analytic approaches. Frameworks for QoL outcome modeling after DBS generally fall into two categories: change-based and end-state models, each with distinct methodological and clinical implications. Change-based models^4,6,7,11,15–17^ quantify improvement relative to baseline and align with the clinical goal of predicting postoperative benefit. However, they are statistically prone to mathematical coupling and regression to the mean, because baseline values are embedded within both predictor and outcome terms^18,19^. Consequently, such models often suggest that patients with poorer baseline QoL show greater postoperative improvement, a pattern that may reflect analytic dependency rather than genuine differential treatment response. End-state models^5,13,14^ circumvent this coupling by using postoperative QoL as the dependent variable, yet they make it difficult to disentangle baseline functioning from the treatment effect, since postoperative QoL remains strongly influenced by preoperative status. Consequently, these models tend to favor patients who already perform well before surgery and provide limited insight into which individuals are likely to experience substantial DBS-related benefit, thereby reducing their utility for preoperative decision support.

The present study adopts an alternative framework that combines the conceptual strengths of both approaches: (1) by defining change as externally anchored and categorical rather than continuous, and (2) by incorporating multimodal predictors to distribute explanatory variance across independent domains.

(1) Applying minimal clinically important difference (MCID) thresholds, i.e., externally validated criteria independent of baseline scores^20–23^, reduces coupling artifacts and aligns the model with clinically meaningful, patient-centered outcomes. Although MCID classification is applied at the individual level, it derives from group-based validation studies, constituting an externally anchored endpoint rather than a direct mathematical transformation of baseline and postoperative scores^19^. This approach minimizes, though does not entirely eliminate, the coupling effect inherent in continuous change-score models while focusing on change that is clinically perceptible^19,22^.

(2) Incorporating a multimodal feature set spanning demographic, motor, cognitive, and affective domains^24,25^ further mitigates analytic dependency and unmeasured confounding arising from incomplete representation of interacting factors^26^. This design distributes explanatory variance across independent dimensions^27^, preventing baseline QoL from dominating the model^28^. Moreover, by analyzing preoperative PDQ-39 subscores rather than the global summary index, the analysis reduces aggregation bias and partial circularity with the postoperative PDQ-39 total. When the same composite score is used at both time points, shared measurement structure can inflate associations between pre- and postoperative values. Using domain-specific subscores preoperatively while retaining the total postoperative score limits this dependency, preserves meaningful variance across QoL dimensions, and allows each baseline domain to contribute independently to outcome prediction^14,19^.

This modified change-based design aims to provide a biologically grounded and clinically interpretable framework for predicting meaningful improvement, addressing the question most relevant to patients and clinicians: which individuals with PD are likely to experience a perceptible QoL benefit after DBS?

**Fig. 1:**
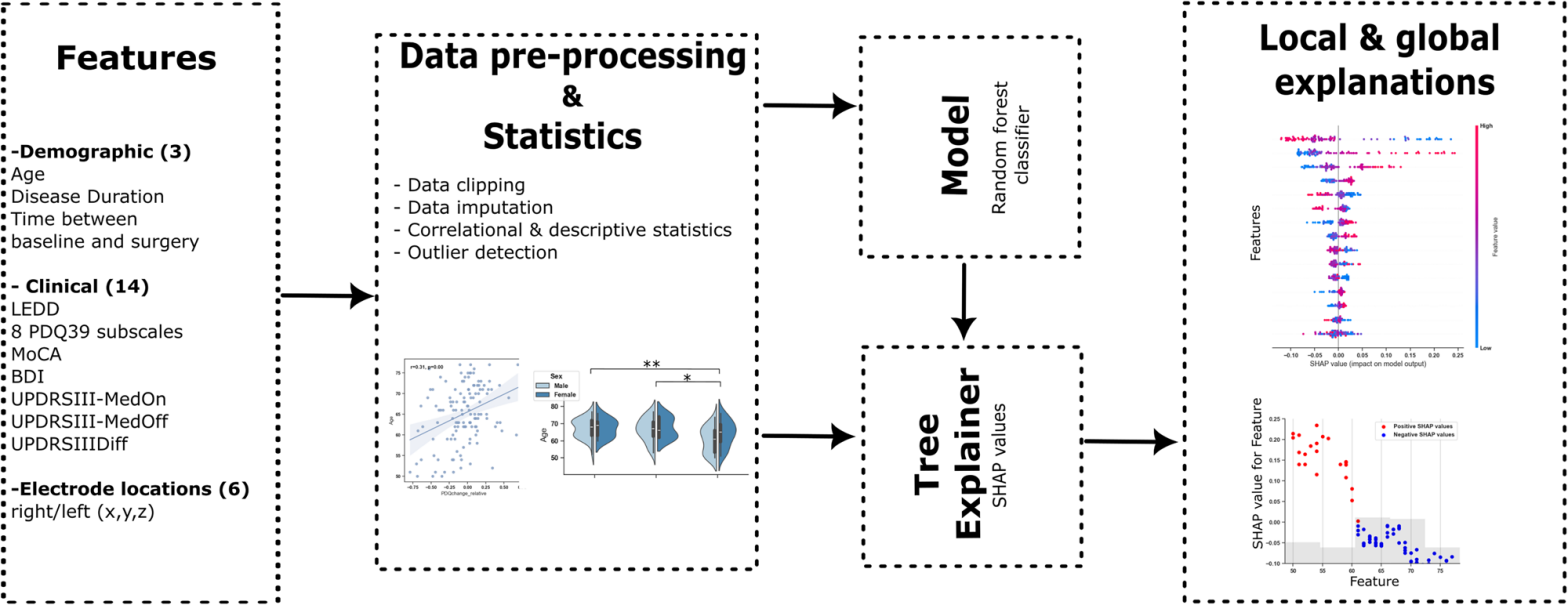
Framework for analysis and model development. Schematic of the analytical workflow, including feature selection, data preprocessing, model training, validation, and interpretation.

Finally, recent evidence indicates that electrode position within the STN influences both motor and non-motor outcomes^29–33^. However, few studies have integrated neuroimaging-derived electrode localization with comprehensive patient-reported and clinical data to explain variability in QoL outcomes^8^. To address this gap and extend our prior work^8^, we apply explainable machine learning to a multimodal dataset that combines demographic characteristics, motor-related measures, and electrode coordinates reconstructed from imaging with cognitive and affective assessments, including all PDQ-39 subscores (Fig. 1). By integrating externally anchored metrics of meaningful change, this multidimensional framework addresses methodological variability across prior studies and generates clinically actionable predictions that support individualized counseling and precision neuromodulation in Parkinson’s disease.

## Results

### Clinical and quality-of-life outcomes after DBS

**Fig. 2:**
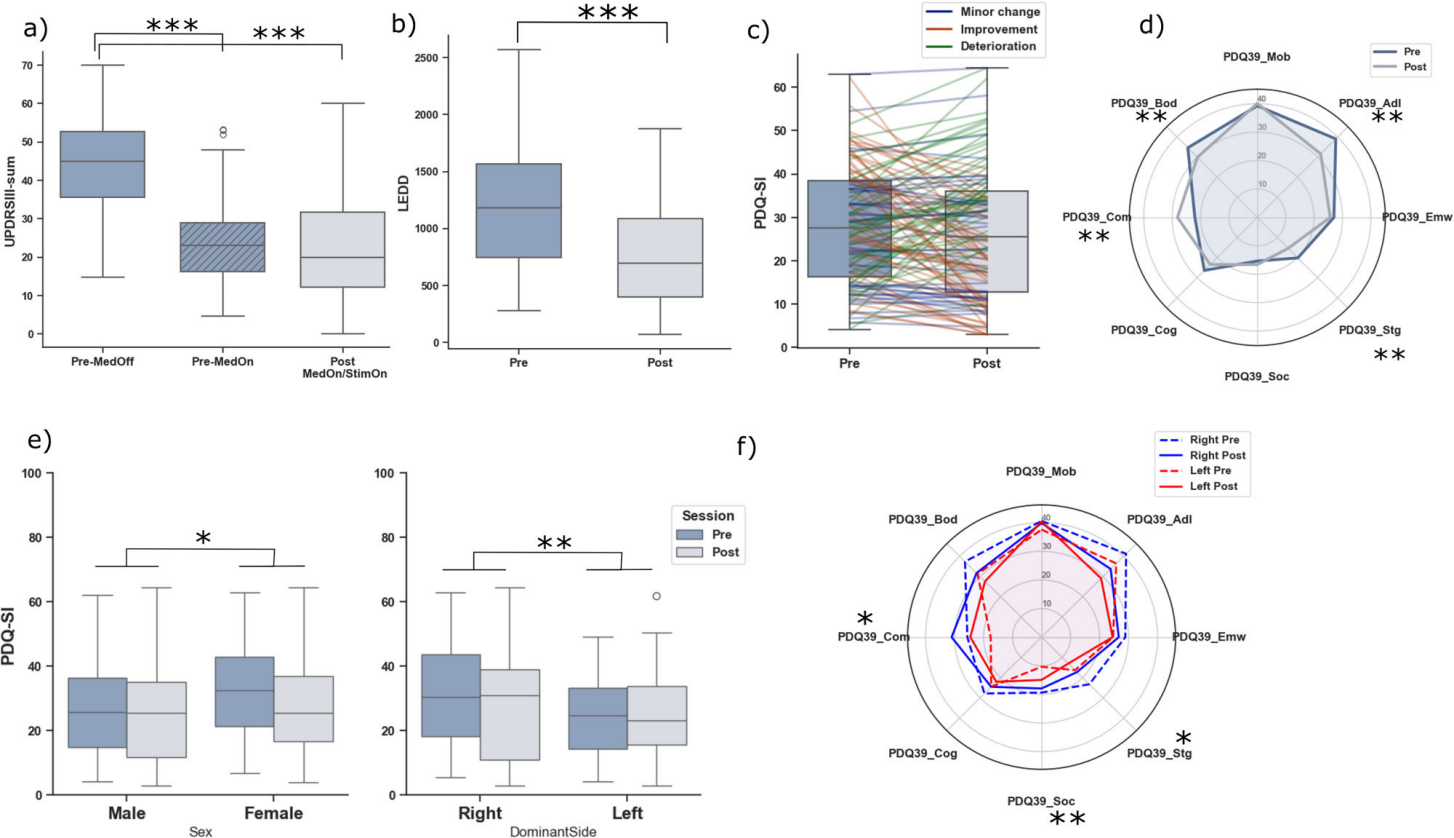
Clinical and quality-of-life outcomes after DBS. **a** UPDRS III scores: motor performance off medication (pre-MedOff), on medication (pre-MedOn), and on medication with DBS stimulation (Post-MedOn/StimOn). Both preoperative medication and postoperative stimulation significantly improved UPDRS III scores (both *p* < 0.001), corresponding to a postoperative therapy response exceeding a 50% reduction in MDS-UPDRS III. **b** Levodopa-equivalent daily dose (LEDD): mean reduction of 53.9% after DBS (*p* < 0.001). **c** PDQ-39 Summary Index (PDQ-39_SI): mean quality-of-life gain of 5.89% after DBS (*p* = 0.27). Of all patients, 36.3% (*n* = 49) improved clinically meaningful, 28.9% (*n* = 39) showed minor change, and 31.1% (*n* = 42) declined. **d** PDQ-39 subscales: significant improvement in activities of daily living, stigma, bodily discomfort (all *p* < 0.01), and significant deterioration in communication (*p* < 0.01). **e** PDQ-39_SI by sex and symptom-dominant side: women and right-dominant patients reported greater quality-of-life burden both before and after surgery (*p* < 0.01, *p* < 0.05). **f** PDQ-39 subscales by dominant side: right-dominant patients had greater burden in stigma, communication (both *p* < 0.05), and social support domains (*p* < 0.01).

An overview of motor, medication, and patient-reported outcomes is presented in Fig. 2. DBS produced robust motor benefits: both Levodopa alone (“Pre-MedOn”) and Levodopa combined with stimulation (“Post-MedOn/StimOn”) significantly improved MDS-UPDRS III motor scores compared with the MedOff condition (*p* < 0.001, Wilcoxon signed-rank test; Fig. 2a). The mean postoperative response exceeded a 50% reduction in motor symptoms. Levodopa-equivalent daily dose (LEDD) decreased by about 40% relative to baseline (*p* < 0.001; Fig. 2b).

While overall quality of life showed only a modest improvement of 5.9% (*p* = 0.27; Fig. 2c), subscale analysis revealed significant postoperative changes across several PDQ-39 domains: activities of daily living (*p* < 0.01), stigma (*p* = 0.003), and bodily discomfort (*p* = 0.01) improved, whereas communication deteriorated (*p* = 0.001; Fig. 2d). On average, the same active contact was maintained for 220 days before follow-up. Over this interval, changes in stimulation amplitude were small, with mean absolute changes of 0.19 ± 0.23 mA in the left hemisphere and 0.15 ± 0.22 mA in the right hemisphere, indicating that postoperative PDQ-39 assessment was generally performed under stable stimulation settings.

A three-way mixed ANOVA showed main effects of sex and symptom-dominant side on overall quality of life across pre- and postoperative assessments (*p* = 0.006 and *p* = 0.045). Females reported poorer quality of life than males (Fig. 2e, left panel). Patients with right-dominant symptoms reported poorer quality of life than patients with left-dominant symptoms (Fig. 2e, right panel), and greater burden for stigma (*p* = 0.01), social support (*p* = 0.006), and communication (*p* = 0.004; Fig. 2f).

### Feature correlations and MCID-based grouping

**Fig. 3:**
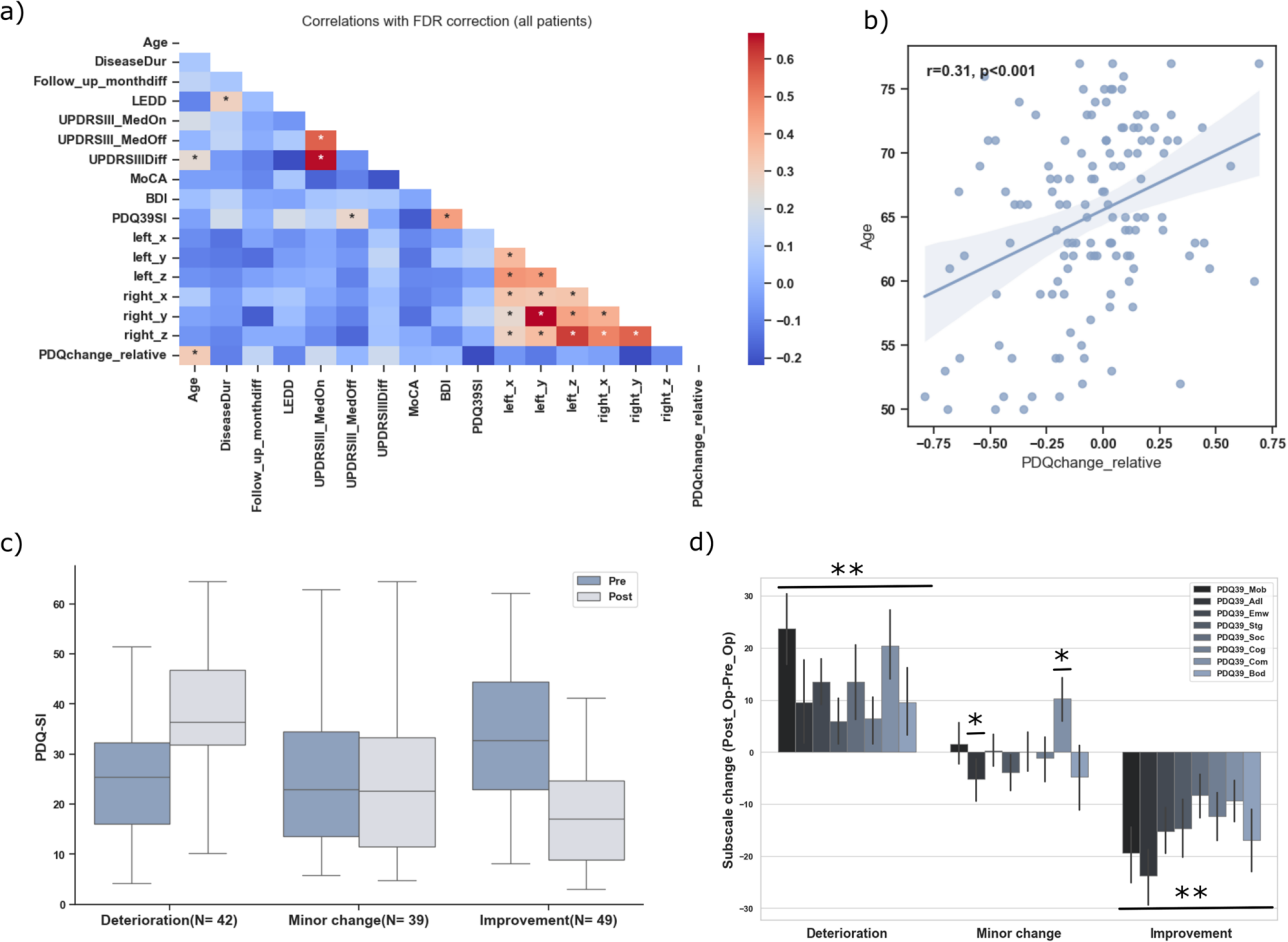
Correlations and PDQ-39 changes after DBS. **a** Heatmap of baseline feature correlations with relative PDQ-39 changes, corrected for multiple comparisons (FDR). Red shading indicates positive correlations, blue shading indicates negative correlations, and intensity reflects magnitude. Significant correlations are marked by asterisks. The left x-coordinate sign was inverted to match the other axes. **b** Scatter plot of age versus PDQ-39_SI change: younger patients showed greater quality-of-life improvement (*p* < 0.001). **c** Patient grouping by minimal clinically important difference (MCID): classification into deterioration (*n* = 42), minor change (*n* = 39), and improvement (*n* = 49). **d** Normalized PDQ-39 subscales with significant group differences: Improvement and deterioration groups changed across all domains (all *p* < 0.01); the minor-change group improved in activities of daily living but worsened in communication (both *p* < 0.05).

Quality-of-life gains declined with increasing age (*p* < 0.001), indicating that younger patients improved more (Fig. 3a, b). UPDRS III scores in the “ON” and “OFF” medication states correlated strongly (*p* < 0.001). Baseline quality of life correlated with both motor function and mood (UPDRS III_MedOn, *p* = 0.01; BDI, *p* < 0.001), showing that patients with greater preoperative impairment tended to be more depressed and have worse motor control. All electrode coordinates were strongly intercorrelated, reflecting symmetrical implantation and a consistent effect of brain size.

**Fig. 4:**
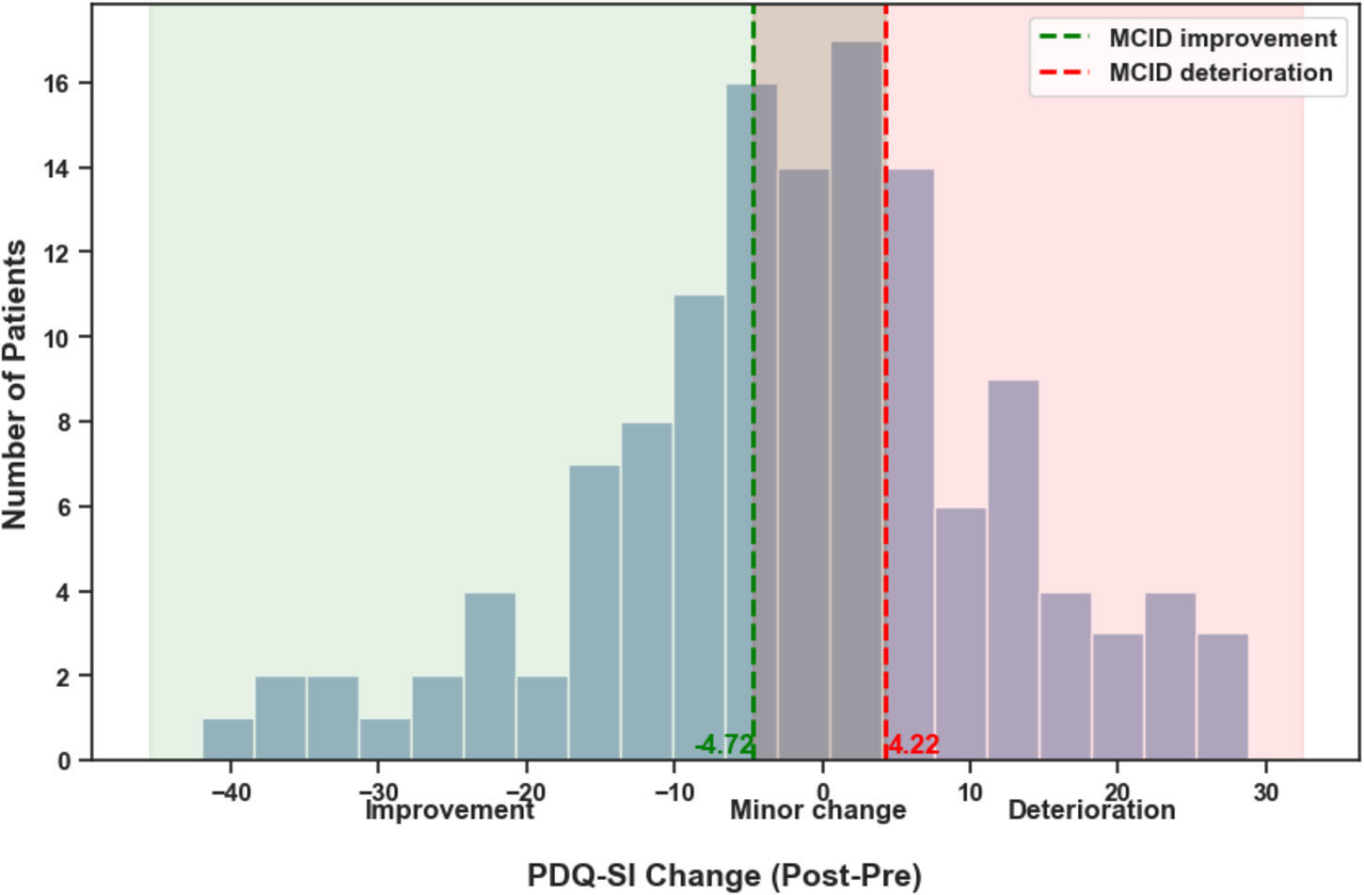
Distribution of individual PDQ-SI change scores and outcome thresholds. Histogram of PDQ-SI change scores across all patients, calculated as postoperative minus preoperative values. Negative values indicate improvement and positive values indicate deterioration. Dashed vertical lines mark the minimal clinically important difference (MCID) thresholds used to define clinically meaningful improvement (green, −4.72) and deterioration (red, 4.22). Patients with change scores between these thresholds were classified as minor change. Shaded regions illustrate the three resulting outcome categories.

Based on MCID thresholds, 49 patients (38%) showed a meaningful improvement, 39 (30%) had minor changes, and 42 (32%) deteriorated (Fig. 3c). Subscale patterns diverged: the improvement group gained across all QoL domains, the deterioration group also declined across all QoL domains, and the minor-change group showed gains in daily living but reduced communication performance (Fig. 3d). Figure 4 additionally illustrates the distribution of individual PDQ-SI change scores across all subjects, with the applied MCID thresholds indicated.

### Baseline predictors of postoperative improvement

**Fig. 5:**
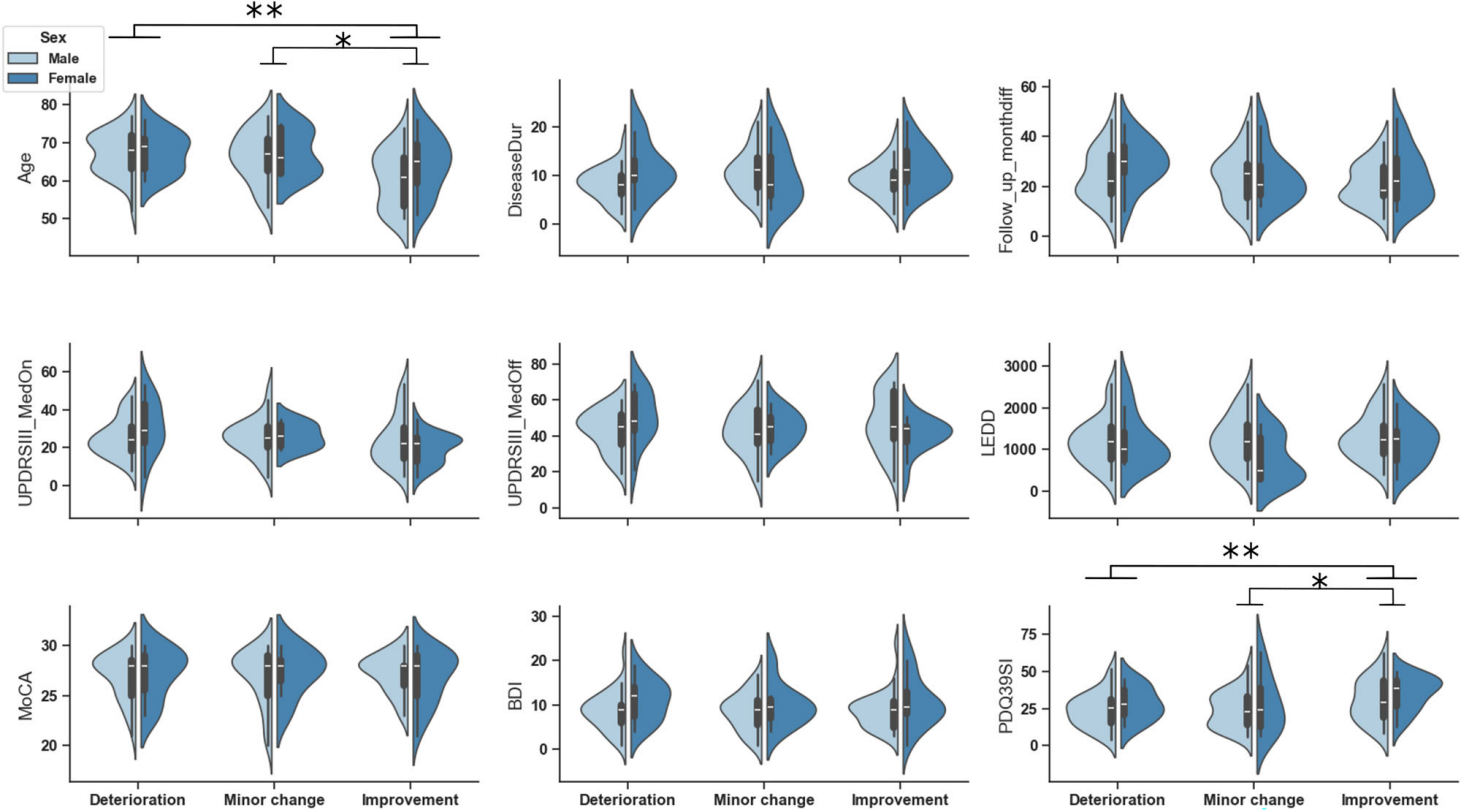
Baseline characteristics by patient group. Distributions of baseline clinical measures (e.g., age, PDQ-39_SI, etc.) in the deterioration, minor-change, and improvement groups. Significant differences are indicated (*p < 0.05, **p < 0.01). The improvement group was significantly younger and reported greater baseline quality-of-life burden than the other groups.

Baseline characteristics differed across MCID categories (Fig. 5). Two-way ANOVA (sex × PDQ group) revealed significant effects of age and baseline quality of life (*p* < 0.001 and *p* = 0.02). Post hoc Tukey tests confirmed that the improvement group was younger and reported poorer preoperative quality of life than both the deterioration (*p* = 0.003; *p* = 0.01) and minor-change groups (*p* = 0.01; *p* = 0.02). Patients with greater preoperative impairment therefore achieved larger postoperative gains.

### Sex-related differences

**Fig. 6:**
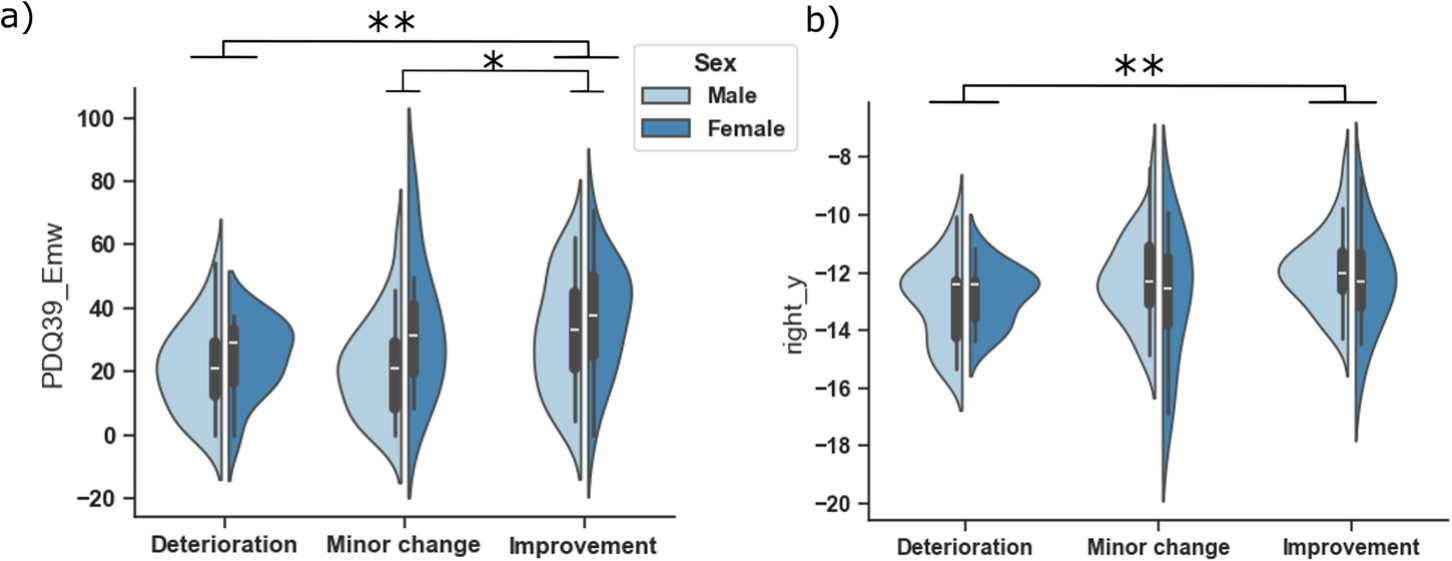
Emotional well-being and electrode placement as determinants of postoperative quality-of-life heterogeneity. **a** Preoperative PDQ-39 emotional well-being burden (PDQ-39_Emw) across MCID-based outcome groups. The improvement group showed greater baseline emotional burden than the other groups (*p < 0.05, **p < 0.01). **b** Right-hemisphere active contact positions along the anterior-posterior axis across MCID-based outcome groups. Patients in the improvement group had more anterior contact locations than those in the deterioration group (**p < 0.01). These panels are shown together because both features emerged from downstream analyses as determinants of postoperative QoL heterogeneity, in addition to age.

Among baseline measures, only disease duration showed a sex effect (*p* = 0.03), with females having longer disease duration (*p* = 0.044). Emotional well-being differed by both group and sex (*p* = 0.005 and *p* = 0.03): females reported greater emotional burden than males (*p* = 0.01), and patients in the improvement group showed the highest preoperative emotional strain (*p* ≤ 0.01; Fig. 6a). Stigma and bodily discomfort also showed sex effects (both *p* < 0.05), indicating greater baseline burden in females.

### Electrode location

**Fig. 7:**
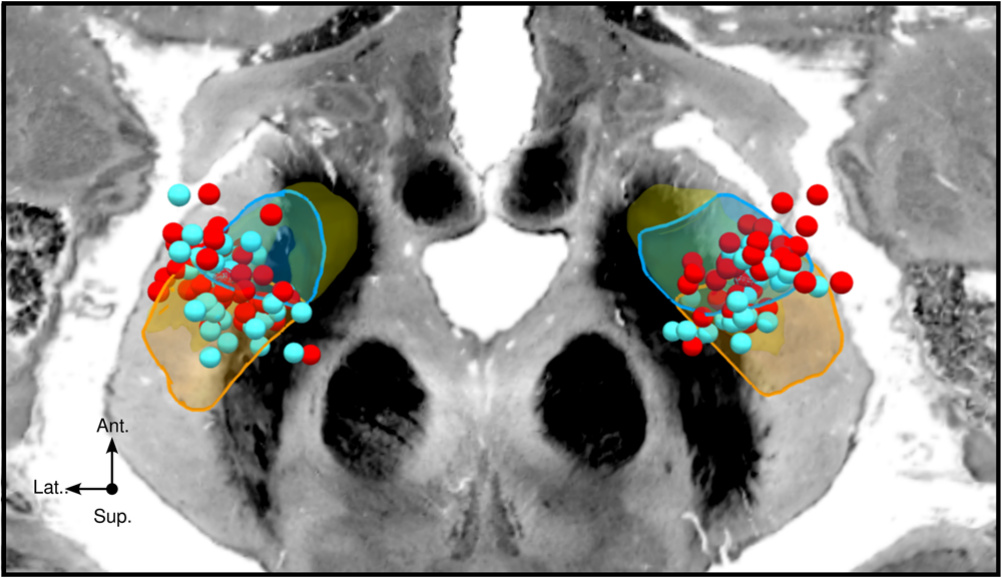
Spatial distribution of active contacts across outcome groups. The spatial distribution of active contacts is shown relative to the motor (orange), associative (blue), and limbic (yellow) subdivisions of the STN derived from the DISTAL atlas (Ewert et al.^68^). Each sphere represents one active contact, with clinically meaningful improvers shown in red and deteriorators in cyan. Contacts in the improvement group were located, on average, slightly anterior to the atlas-based motor-associative border (0.53 ± 1.37 mm), whereas contacts in the deterioration group were located, on average, slightly posterior to this border (−0.18 ± 1.15 mm).

Active contact position along the right-hemisphere *y*-axis differed significantly across outcome groups (*p* = 0.02). Patients in the improvement group had more anterior contact positions than those in the deterioration group (*p* = 0.01; Fig. 6b). To provide anatomical context for the anterior-posterior effect, we quantified the distance of active contacts from the atlas-based motor-associative STN border (Fig. 7). Contacts in the deterioration group were located, on average, posterior to the border (−0.18 ± 1.15 mm), whereas contacts in the improvement group were located, on average, anterior to it (0.53 ± 1.37 mm).

### Parkinson’s disease subtype and quality-of-life outcome

**Fig. 8:**
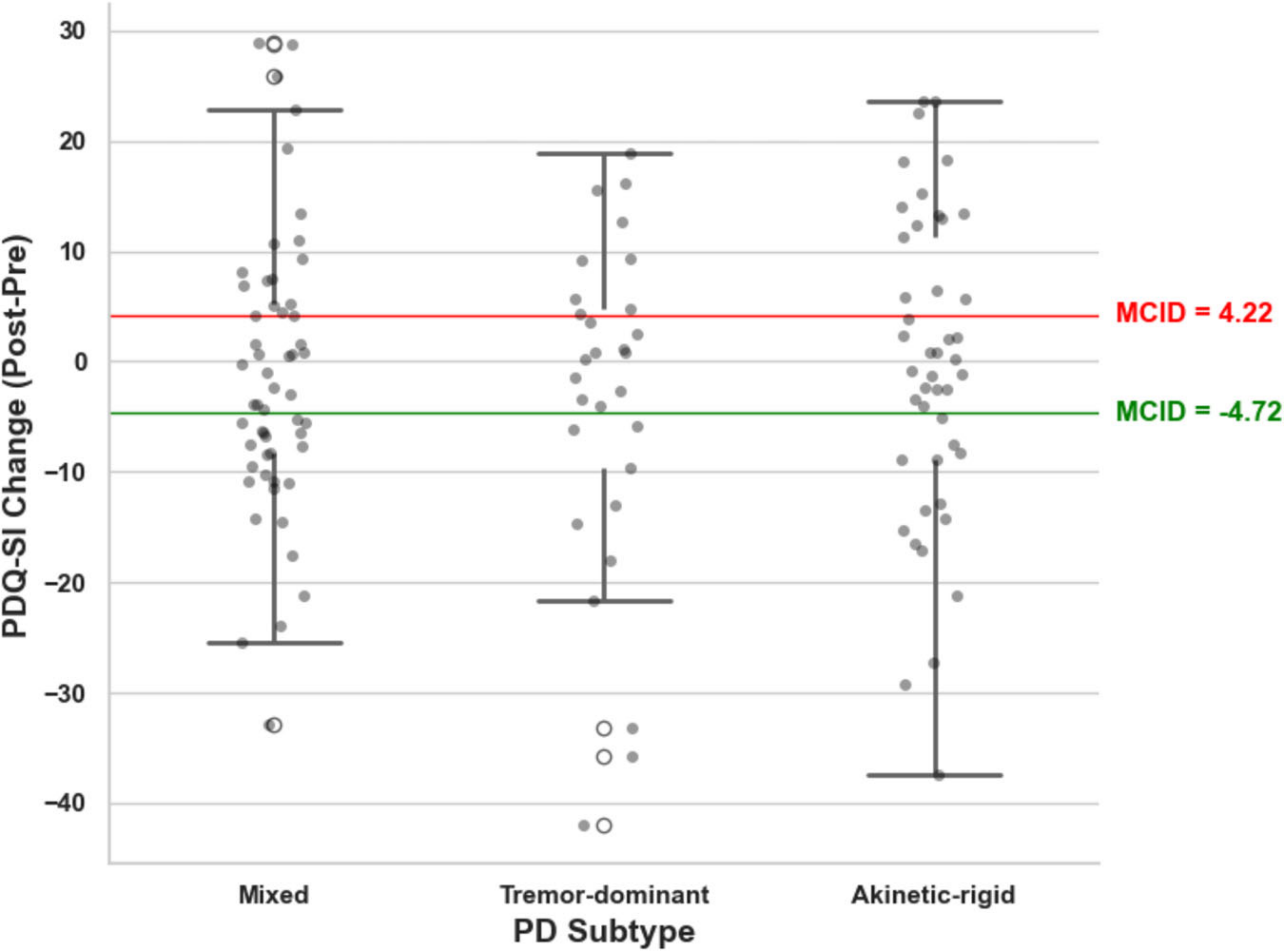
Postoperative PDQ-SI change across Parkinson’s disease subtypes. Individual PDQ-SI change scores, calculated as postoperative minus preoperative values, are shown for patients classified as mixed (*n* = 56), tremor-dominant (*n* = 29), and akinetic-rigid (*n* = 45). Negative values indicate improvement and positive values indicate deterioration. Horizontal lines mark the minimal clinically important difference (MCID) thresholds for clinically meaningful improvement (−4.72, green) and deterioration (4.22, red). No significant difference in postoperative PDQ-SI change was observed across subtypes (Kruskal-Wallis test, *p* = 0.84), indicating that Parkinson’s disease subtype did not significantly contribute to clinically meaningful postoperative quality-of-life change in this cohort.

Parkinson’s disease subtype (tremor-dominant (*n* = 29), akinetic-rigid (*n* = 45), or mixed (*n* = 56)) did not differ across outcome groups (Fig. 8) and did not show a significant association with postoperative change in PDQ-39 Summary Index (Kruskal-Wallis test, *p* = 0.84).

### Predictive modeling of quality-of-life outcomes

A random-forest classifier was trained to distinguish improvement (*n* = 49) from deterioration (*n* = 42) using baseline variables: age, disease duration, LEDD, PDQ-39 subscales, MoCA, BDI, UPDRS III scores (MedOn, MedOff, difference ratio), electrode coordinates, and follow-up interval.

The dataset was split into 90% training/validation and 10% testing subsets. Five-fold cross-validation yielded training accuracy of 95% and validation accuracy of 69%. On the held-out test set, accuracy was 70% (AUC of 0.70, sensitivity of 80%, specificity of 60%, positive predictive value of 70%, negative predictive value of 72%). Adding PD subtype as an additional feature did not improve model performance, with validation accuracy decreasing from 70 to 65%.

### Feature importance and SHAP analysis

**Fig. 9:**
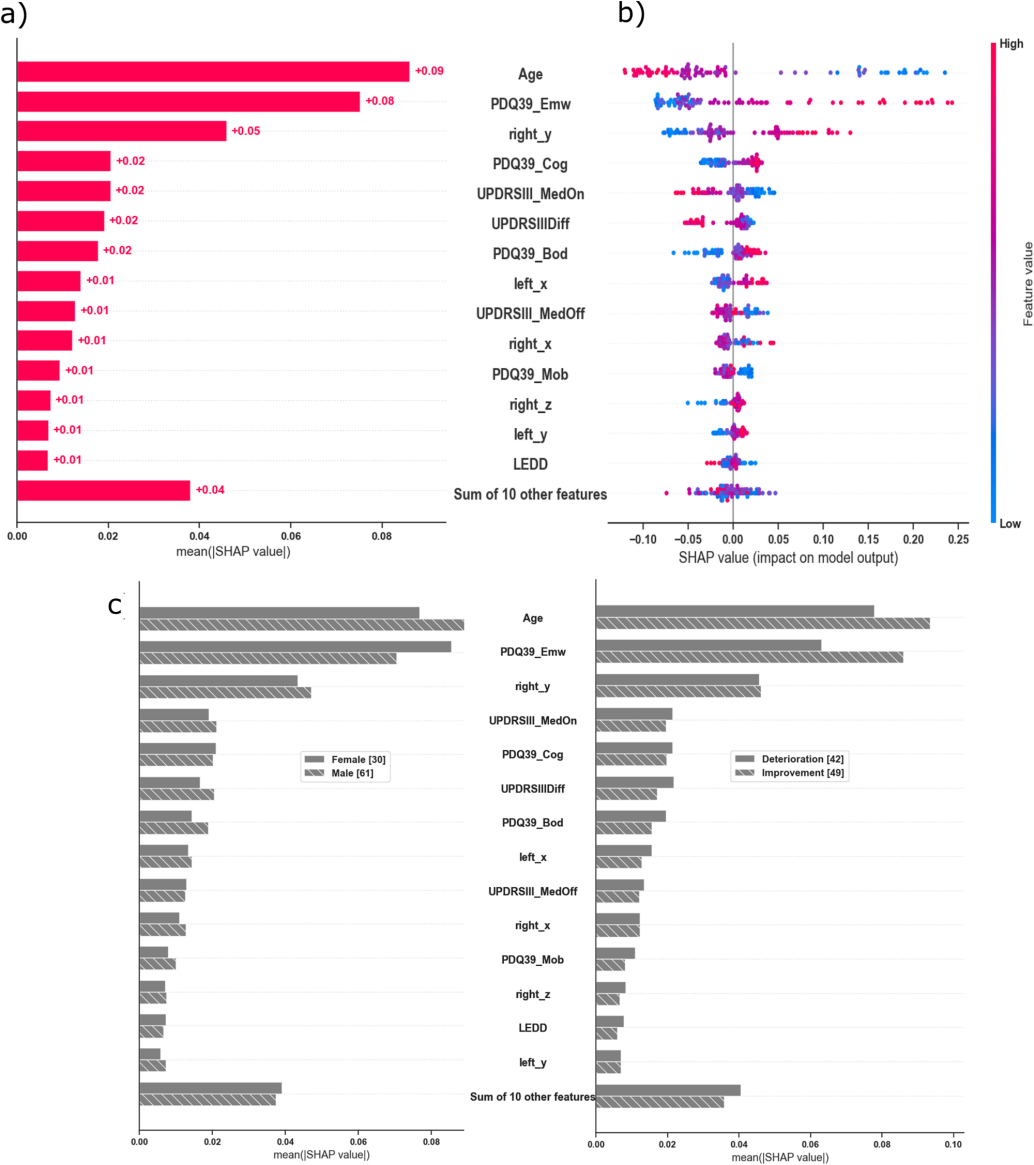
SHAP-based feature analysis. **a** Feature importance: mean SHAP values ranking the contribution of each variable to the classifier. **b** SHAP value distribution: global feature impact on model output for predicting clinically meaningful QoL improvement. Each dot represents a patient, with color indicating the feature value (red, high; blue, low). The position along the *x*-axis reflects the SHAP value, indicating the direction and magnitude of each feature’s influence on the model prediction. Positive SHAP values shift the model output toward predicting improvement, whereas negative values indicate a contribution toward predicting deterioration. The horizontal spread of points shows interindividual variability in how strongly each feature influences prediction. **c** Subgroup analysis: feature importance by sex and by outcome (improvement vs. deterioration). Age and PDQ-39_Emw were the top predictors of improvement, age being more influential in males and PDQ-39_Emw in females.

Feature-importance analysis identified age, preoperative emotional burden, and the right-hemisphere y-coordinate of the electrode as the most influential predictors (Fig. 9a). Additional variables, including UPDRS III_MedOn and cognitive function, contributed less. Left-hemisphere electrode coordinates and Parkinson’s disease subtype did not emerge as relevant predictors in the SHAP-based feature importance analysis. SHAP visualization (Fig. 9b) showed that younger age, greater emotional burden before surgery, and more anterior right-hemisphere electrode positions were associated with a higher likelihood of postoperative improvement. Feature relevance varied by sex: age contributed more strongly in males, while emotional burden had greater predictive weight in females (Fig. 9c, left). Both features were decisive for identifying patients who improved (Fig. 9c, right).

### Feature thresholds derived from SHAP dependence plots

**Fig. 10:**
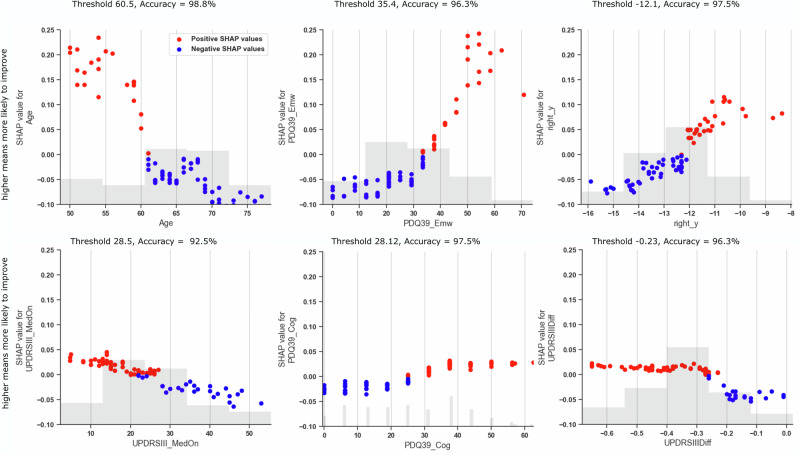
Cut-off values of key features influencing model prediction. Dependence plots showing the relationship between feature values and their contribution to model predictions for the six most influential features. Red = positive, blue = negative SHAP contributions. Thresholds were derived using a linear SVM and validated by fivefold cross-validation. Predictors of postoperative quality-of-life improvement included: • age < 60.5 years • greater preoperative emotional-well-being burden (PDQ-39_Emw > 35.4) • more anterior right-hemisphere electrode position (y > −12.1 mm) • better preoperative motor function (UPDRS III_MedOn < 28.5) • levodopa response >23% • greater preoperative cognitive burden (PDQ-39_Cog > 28.12).

Dependence plots (Fig. 10) identified clinically interpretable cut-offs for factors associated with a higher likelihood of postoperative improvement:

- Age below 60.5 years
- Greater preoperative emotional burden (PDQ-39_Emw > 35.4)
- More anterior right-hemisphere electrode position (y > −12.1 mm)
- Better preoperative motor function on medication (UPDRS III_MedOn < 28.5)
- Levodopa response above 23%
- Greater preoperative cognitive burden (PDQ-39_Cog > 28.12)

## Discussion

This study addressed the marked heterogeneity of QoL outcomes following DBS for PD. Although DBS consistently improves motor function, patient-perceived benefit remains highly variable. The present study sought to clarify this variability by integrating demographic, motor, affective, cognitive, and anatomical factors into an explainable predictive framework anchored on clinically meaningful QoL change. This multidimensional approach provides complementary insights into the mechanisms underlying patient-perceived benefit and allows disentangling treatment-responsive domains from those that remain stable despite symptomatic improvement.

Three independent determinants of postoperative QoL improvement were identified: younger age, greater disease-related emotional burden, and an electrode location at the motor-associative transition in the right subthalamic nucleus. These dimensions—biological, psychological, and anatomical—jointly account for the heterogeneity of individual treatment responses. The random forest model achieved moderate discrimination (AUC = 0.70) with balanced positive and negative predictive values around 70%, providing an explanatory rather than clinically predictive level of performance, and reflecting the multifactorial nature of QoL outcomes. The model therefore serves primarily as a hypothesis-generating framework, highlighting which features contribute most to postoperative variability and informing strategies for individualized counseling and future research design. Accordingly, these findings are not intended to redefine patient selection criteria, intraoperative targeting strategies, or postoperative benchmarks of success, but rather to clarify why patients with comparable motor benefit may nevertheless differ substantially in their perceived postoperative QoL. In this sense, the present framework is best understood as supporting preoperative counseling and expectation management. Postoperative QoL should therefore be viewed as arising from the combined effects of motor improvement, medication reduction, electrode location, demographic characteristics, and non-motor as well as psychosocial factors, rather than from any single predictor in isolation.

Previous studies have examined the influence of age on QoL outcomes after subthalamic DBS, with most patient cohorts averaging around 60 years at the time of surgery^9,34^. While significant QoL improvements have been reported across age groups^34^, several studies found that higher age was associated with smaller PDQ-39 summary index gains or weaker improvement across subscores such as stigma, activities of daily living, mobility, cognition, and communication, whereas others observed no significant age effect^9^. To derive more interpretable insights, later analyses applied refined analytic approaches using the PDQ-8 short form. One study stratified patients into three age groups (≤59, 60–69, ≥70 years) and found large, moderate, and small effect sizes for QoL improvement, respectively, with broader domain gains in the youngest group^15^. Another analysis used a distribution-based threshold of one-half the baseline standard deviation and identified both younger age at surgery and greater baseline non-motor burden, characterized by anhedonia and concentration difficulties, as predictors of greater QoL improvement at 36 months^6^.

In the present work, we extended these findings by applying a patient-centered, data-driven approach grounded in the externally anchored MCID, derived from patient-reported global impression of change and validated against distribution-based estimates^23^. Combining this MCID framework with random-forest modeling, SHAP-based feature attribution, and SVM-derived cut-off thresholds yielded clinically interpretable results, indicating that patients aged 60 years or younger have a markedly higher likelihood of postoperative QoL improvement. This refines the broader ~70-year threshold identified in earlier work that did not incorporate MCID-based calibration^8^. While derived from a single-center experience, these findings should be regarded as exploratory and intended to inform, not define, clinical decision-making. They emphasize an optimal therapeutic window rather than a strict eligibility limit. Notably, the identified age aligns closely with the upper boundary of the EARLYSTIM cohort, where the mean age in the DBS group was 52.9 ± 6.6 years^35^, supporting the notion that maximal QoL benefit occurs when stimulation is initiated while functional and neural reserves remain substantial.

Preoperative emotional well-being emerged as the strongest non-demographic predictor of postoperative QoL. Patients reporting greater disease-specific emotional burden before surgery experienced the most pronounced overall QoL improvements, particularly in the domains bodily discomfort, stigma and activities of daily living, even though the emotional well-being domain itself did not significantly change after DBS (Fig. 2d). Moreover, the communication domain worsened postoperatively, a pattern consistent with the known phenomenon of residual or stimulation-induced speech impairment after DBS^36^. This apparent dissociation indicates that DBS may improve the QoL domains related to the functional and social consequences of the disease rather than the disease-specific emotional strain that may persist even after improvement of motor function. Patients who were most emotionally affected by their disease perceive the greatest relief once DBS reduces the practical limitations feeding that distress, while the underlying emotional stance toward the illness remains stable. This interpretation aligns with response-shift theory, which posits that patients recalibrate QoL appraisal following major medical interventions^37^. Comparable dissociations between affective subscales and global QoL improvement have been reported in epilepsy, multiple sclerosis, stroke recovery, and cardiac surgery, where functional restoration enhances life satisfaction but leaves chronic disease-related worry largely unchanged^38–41^. The persistence of emotional burden despite improvement in other domains therefore may reflect a trait-like illness representation rather than treatment resistance.

Interestingly, general depressive symptom severity, as measured by the BDI, did not predict postoperative QoL changes^42^. The BDI quantifies non-specific depressive symptoms such as sadness or anhedonia, whereas the PDQ-39 emotional well-being domain captures PD-specific emotional distress rooted in daily functional experience^43^. The strong predictive value of the PDQ-39 emotional index therefore suggests that disease-specific affective appraisal, rather than general mood disturbance, determines perceived benefit after DBS. This distinction emphasizes the importance of using disease-contextualized affective measures in preoperative evaluation.

Recent work reported an apparently opposite pattern using the Hospital Anxiety and Depression Scale (HADS)^17^. Higher preoperative depression and anxiety scores on the HADS predicted poorer postoperative QoL, as measured by the PDQ-8. However, this finding is complementary rather than contradictory. The HADS and BDI quantify general psychopathological affect, reflecting broader emotional states such as hopelessness, anhedonia, or anxiety that often represent stable psychological traits or comorbid affective disorders^42^. In contrast, the PDQ-39 emotional well-being subscale captures disease-specific emotional distress that arises from the lived experience of PD, encompassing worry about progression, frustration with communication, or embarrassment in public^43^. These items anchor emotional state within the context of motor disability, representing a modifiable affective response rather than a fixed mood disorder. Consequently, broad affective load measured by the BDI or HADS may constrain postoperative adaptation, while PD-specific emotional distress identified by the PDQ-39 highlights patients whose global QoL is particularly sensitive to relief from functional limitations. This interpretation reconciles the differing directions of association across instruments and reinforces the value of disease-specific affective metrics for predicting subjective benefit following DBS.

Cognitive measures further support this affective model. Objective cognitive performance (MoCA) did not contribute to QoL prediction, whereas the PDQ-39 cognitive domain did. Prior validation work showed that the PDQ-39 cognition domain correlates more strongly with depression and anxiety than with objective neuropsychological performance^44^. Thus, it represents subjective cognitive-affective complaints rather than measurable cognitive decline. Its predictive relevance in our dataset reinforces that affective self-perception, not cognitive ability, drives postoperative QoL trajectories.

The location of electrodes within the STN also emerged as a key determinant of QoL outcomes. Specifically, our analysis revealed that positions along the anterior-posterior axis of the STN, particularly (but not only) in the right hemisphere, significantly influenced postoperative QoL changes. This hemispheric difference may be related to both cognitive and affective mechanisms previously reported for the right STN^45,46^. In the present study, active contact locations anterior and posterior to *y* = −12 in standard MNI space were associated with positive and negative effects, respectively. Three-dimensional reconstructions in standard MNI space further showed that active contacts located more superiorly, laterally, and centrally within the STN, near the transition between its motor and associative subregions, were associated with greater postoperative QoL improvement (Fig. 7). These results complement prior research indicating that active contact positions above *z* = −7 in standard MNI space had positive effects on QoL^8^. Together, these findings suggest the motor-associative transition zone of the STN as a “sweet spot” for optimizing QoL improvements, consistent with previously reported motor “sweet spots”^29,30,47–50^. At the same time, this observation should not be interpreted as a recommendation to shift surgical targeting away from regions optimized for motor benefit. Rather, it provides anatomical context for postoperative heterogeneity within the vicinity of established targets and may inform future studies on how directional steering or field-shaping approaches influence patient-perceived outcome without compromising motor efficacy.

However, as summarized previously^8^, research on the influence of electrode placement on non-motor outcomes and QoL remains inconsistent^15,31,32^. Earlier studies suggested that more anterior, medial, and ventral electrode locations were associated with better outcomes in terms of non-motor symptoms and QoL^15^. Subsequent work from the same group confirmed this trend, linking improvements in mood, attention, memory, and sleep to QoL enhancements following DBS^31^. These improvements appeared to depend on electrode placement closer to the ventral or lower STN border, regions typically not targeted for motor improvements^29,30,47,48,50,51^. Conversely, recent findings from the EARLYSTIM cohort suggest that the optimal region for QoL improvements lies posterior and superior to the motor “sweet spot,” near the upper STN border^32^.

One possible explanation for these discrepancies is that postoperative QoL improvements may arise from distinct mechanisms. Stimulation along the dorsolateral STN border, as suggested by the present findings, appears to enhance QoL primarily through functional and social domains such as bodily discomfort, stigma, and activities of daily living. In contrast, stimulation along the ventromedial border has been linked to improvements in non-motor and affective domains, including mood, attention, and sleep^31^. This functional segregation implies that QoL outcomes reflect a balance between motor-associative and limbic circuit modulation. Future research should explore dual-target or directionally optimized stimulation paradigms^33,52^ that engage both territories to achieve broader, more stable QoL gains. Given that most patients in our cohort were stimulated in ring mode and no patient received multiple independent current control, the present analysis used active contact location as a pragmatic proxy for the effective stimulation site. More advanced modeling approaches, including volume of tissue activated estimation and current steering frameworks, may provide a more precise characterization of stimulation fields in future studies^53^.

In our cohort, female patients showed longer disease duration and greater preoperative burden in the emotional well-being, stigma, and bodily discomfort domains. These sex-related differences are consistent with previous studies demonstrating that women with Parkinson’s disease experience higher affective and psychosocial strain despite comparable motor severity. Cross-sectional and longitudinal analyses have shown that female patients report poorer QoL, particularly in emotional well-being, bodily discomfort, and stigma^10,54^. In multicenter DBS cohorts, women also present with longer disease duration and higher baseline bodily discomfort, yet demonstrate smaller postoperative improvements in emotional domains^55^. Together, these data suggest that women may enter surgery with a heavier psychosocial load that moderates perceived benefit even when motor outcomes are comparable. Incorporating sex-specific affective and social predictors into future models may improve individualized patient counseling and outcome forecasting.

Symptom-dominant side was included in the initial analyses as a clinically relevant measure of disease laterality. In our cohort, patients with right-dominant symptoms reported poorer overall QoL and greater burden in stigma, social support, and communication. This pattern is of interest because motor symptom asymmetry in PD has been linked not only to lateralized motor presentation, but also to differences in non-motor outcomes that may shape patient-perceived disease burden and QoL^56^. In particular, the predominance of differences in stigma, social support, and communication, rather than in domains more directly related to manual daily function, suggests that this effect is less likely to be explained primarily by dominant-hand involvement. This interpretation is also consistent with a systematic review^56^, which found that right-dominant motor symptoms, reflecting predominant left-hemispheric involvement, are more consistently associated with cognitive decline and increased dementia risk. The subscale pattern observed here therefore appears more compatible with a broader cognition-related impact on communication and social functioning than with a purely motor explanation. Since handedness was not systematically documented in our dataset, this interpretation remains exploratory and should be examined in future cohorts with systematic handedness assessment.

By contrast, PD subtype did not contribute to postoperative QoL change in our cohort and did not improve model performance, despite its established clinical relevance for symptom characterization and treatment planning. This suggests that subtype, while important for describing disease phenotype, may contribute less to patient-reported postoperative QoL heterogeneity than age, emotional burden, and electrode location in the present setting.

The relatively small sample size limited the application of more advanced clustering methods and predictive modeling, yet the current framework highlights the utility of combining clinical and patient-reported data to extract actionable insights. The absence of postoperative StimOn/MedOff assessments limits the ability to isolate the specific contribution of DBS independent of dopaminergic medication, and thus prevents a direct evaluation of pure stimulation efficacy and electrode placement effects. In addition, motor complications such as off-time and dyskinesia were not included due to limited availability of UPDRS IV data, although these factors are known to influence patient-reported QoL. Lead localization was based on postoperative CT acquired within 24 h after implantation. Although this follows standard clinical practice at our center, immediate postoperative effects may influence apparent lead orientation. In the present cohort, this limitation is mitigated by the fact that directional stimulation was used only in a small subset of patients.

Although our model classified patients using a single global QoL endpoint, this categorization reflected concordant directional changes across all QoL domains rather than an isolated summary-score effect. Furthermore, QoL was assessed at a single postoperative time point (mean follow-up ~19 months), which does not capture long-term trajectories. This limitation is particularly important in a chronic progressive disorder such as PD. Longitudinal studies have shown that initial improvements after DBS may attenuate over time due to disease progression, highlighting that single time-point measures cannot fully represent the chronic and evolving nature of PD. At the same time, our findings and the recent 5-year INTREPID study data^57^ appear complementary. The significant postoperative changes observed in our cohort (mean age 65 years) occurred in the same domains (activities of daily living, stigma, and bodily discomfort) that showed sustained benefit in the younger INTREPID cohort (mean age 60 years). Since the patients in our cohort with a higher likelihood of improvement were also younger, and within a similar age range (<60.5 years), this raises the possibility that the early favorable pattern identified here may reflect a more durable trajectory. Taken together, these findings raise the possibility that younger patients are more likely to achieve postoperative QoL benefits that may persist long term, whereas older patients may already show early deterioration in these domains, potentially foreshadowing a less favorable longer-term course. These inferences remain speculative and require direct longitudinal validation.

We note that stimulation settings were largely stable for several months before postoperative PDQ-39 assessment, supporting the interpretation that the reported QoL outcomes reflect a relatively steady-state stimulation condition rather than short-term programming effects. However, larger, multicenter datasets incorporating broader variable sets will be needed to confirm the present findings and clarify the complex determinants of postoperative QoL trajectories

In conclusion, this study demonstrates that data-driven integration of demographic, affective, and anatomical factors identifies key predictors of meaningful QoL improvement after DBS, including younger age, greater disease-specific emotional burden, and electrode placement at the motor-associative transition of the STN, providing a path toward more precise patient stratification, individualized counseling, expectation management, and a more nuanced understanding of postoperative QoL heterogeneity.

## Methods

### Patients

This study included 150 patients with Parkinson’s disease (mean age: 65 years (SD 7.1); 44 women and 106 men; total 300 electrode leads). The mean age was 66.3 years (SD 6.6) for women and 64.5 years (SD 7.2) for men. All patients underwent STN-DBS and had complete pre- and postoperative data relevant to the study aims. Fifty of these patients had been included in a previous analysis^8^; methods shared with that study are cited accordingly. The primary outcome measure, the 39-item Parkinson’s Disease Questionnaire (PDQ-39)^43^, was obtained during hospital visits before DBS implantation and at follow-up. The scale provides both the summary index (PDQ-39_SI) and eight normalized domain scores: mobility (PDQ-39_Mob), activities of daily living (PDQ-39_Adl), emotional well-being (PDQ-39_Emw), social support (PDQ-39_Soc), cognition (PDQ-39_Cog), communication (PDQ-39_Com), bodily discomfort (PDQ-39_Bod), and stigma (PDQ-39_Stg). PDQ-39 scores were directionally adjusted for readability: higher raw values indicate poorer quality of life, but all results are described in intuitive terms (improvement = gain in quality of life, burden = poorer baseline status).

All participants underwent DBS implantation after overnight withdrawal of dopaminergic medication. No surgical complications were reported. The study was conducted in accordance with the Declaration of Helsinki and approved by the University Hospital Tübingen Ethics Committee (781/2015B02). The need for informed consent was waived because all data were collected as part of routine clinical care.

### Clinical assessments

Baseline clinical assessments were conducted on average 4 months before surgery (SD 4). A detailed medical history was obtained, including medication, disease duration, symptom-dominant side, and levodopa equivalent daily dose (LEDD). Symptom-dominant side was included as a clinically relevant measure of disease laterality, to test whether baseline asymmetry was associated with patient-reported QoL burden across assessments. Motor function was assessed with the Movement Disorder Society Unified Parkinson’s Disease Rating Scale Part III (UPDRS III) in dopaminergic “ON” and “OFF” states^58^. When only the older UPDRS III version was available, scores were converted^59^. UPDRS IV follow-up scores assessing motor fluctuations and dyskinesia were only available in a small subset of patients and were therefore not included in the analysis. Cognitive function was assessed with the Montreal Cognitive Assessment (MoCA)^60^. When only the Mini-Mental State Examination (MMSE)^61^ was available, total scores were converted to MoCA-equivalent scores using a validated conversion table^62^. Depressive symptoms were measured with the Beck Depression Inventory (BDI-I)^42^ or its revised version (BDI-II)^63^. PD subtype information was also available and patients were classified as tremor-dominant, akinetic-rigid, or mixed for additional analyses. Follow-up assessments were performed on average 19 months after surgery (SD 8). Quality-of-life change was quantified using MCID thresholds for the PDQ-39_SI^23^: improvement = –4.72, deterioration = +4.22. Patients with postoperative PDQ-39_SI reductions ≥4.72 points were classified as improved; those with increases ≥4.22 points were classified as deteriorated; others were considered minor change. Additional outcomes included LEDD reduction and UPDRS III changes (±1 year around the PDQ time point). Baseline features included: sex, age, disease duration, symptom-dominant side, LEDD, all PDQ-39 domain scores, MoCA, BDI, UPDRS III in medication-on and medication-off states (UPDRS III_MedOn and UPDRS III_MedOff), relative motor improvement (UPDRS III_Diff = (UPDRS III_MedOn – UPDRS III_MedOff)/(UPDRS III_MedOn + UPDRS III_MedOff)), active electrode coordinates (left/right x, y, z), and the interval in months between baseline and follow-up (Fig. 1). Postoperative motor assessments in the stimulation-on/medication-off condition (StimOn/MedOff) were not routinely acquired in our clinical workflow and were therefore not available for analysis. To assess stimulation stability, we quantified the duration of unchanged active contacts and changes in stimulation amplitude prior to follow-up.

### Data preprocessing

Feature values were clipped to the range between the 2nd and 98th percentiles to reduce outlier influence. Missing data were imputed using median univariate imputation. Outliers were then excluded based on a twofold interquartile range (2×IQR) criterion for each feature (values < Q1 – 2 × IQR or >Q3 + 2 × IQR). Based on this criterion, 20 patients were excluded due to feature-specific deviations: PDQ-39_Soc (P44); MoCA (P16, P34, P51, P106, P121); BDI (P3, P36, P44, P53, P65, P71, P79, P121); left_y (P20, P29, P34); left_z (P8, P20, P29, P34, P65); right_x (P20, P22, P29, P34); right_y (P20, P29, P34, P68); right_z (P1, P11, P17, P20, P29, P34, P65). After preprocessing, 130 patients remained for statistical and machine-learning analyses (Fig. 1).

### Statistical analysis

Between-subject comparisons were performed using paired *t* tests or Wilcoxon signed-rank tests, depending on data normality (Shapiro-Wilk test). These analyses were applied to the full cohort and to subgroups defined by PDQ-39_SI outcome (improvement, deterioration, or minor change). Three-way mixed ANOVA was used to assess the effects of session (pre- vs. post-surgery), sex, and dominant side on PDQ-39_SI. Two-way ANOVA examined the effect of dominant side on individual PDQ-39 subscales. Two-by-two ANOVA tested interactions between sex and PDQ category for each clinical feature. PD subtype was compared across outcome groups using the Kruskal-Wallis test and was additionally evaluated as an input feature in the predictive model. Post-hoc pairwise comparisons used Tukey’s HSD. For non-normal data, rank transformation was applied before analysis. Spearman’s *ρ* was used for correlation analyses, with significance at *p* < 0.05. Multiple comparisons were corrected using the false discovery rate (FDR) method of Benjamini and Hochberg^64^.

### Random-forest classifier

Classification analyses compared patients who improved with those who deteriorated. A random-forest classifier was implemented to identify preoperative predictors of improvement. Tree-based models were chosen for their robustness on tabular datasets and interpretability^65^. Data were split into 90% training/validation and 10% testing subsets. The classifier was trained using fivefold stratified cross-validation (StratifiedKFold = 5). Hyperparameters, including number of estimators, maximum tree depth, maximum features, and minimum samples per split, were optimized via grid search. The final model was selected based on maximum validation accuracy and evaluated on the independent test set.

### Feature-importance analysis

Feature contributions were quantified using SHapley Additive exPlanations (SHAP)^65^. Shapley values quantify the magnitude and direction of each feature’s effect on individual predictions^66^. Absolute mean Shapley values were visualized as bar plots to represent global feature importance. Beeswarm plots illustrated value distributions and impact direction for individual predictions. Scatter plots of SHAP versus feature values showed relationships at the patient level, where higher SHAP values indicated a greater probability of classification in the improvement group. Cut-off thresholds separating positive and negative SHAP values were determined by training a linear support vector machine (SVM) on each feature’s SHAP-value relationship. Threshold reliability was evaluated using fivefold cross-validation.

### Electrode localization and visualization

Electrode trajectories were reconstructed from patient-specific postoperative imaging using the Lead-DBS pipeline^67^, whereas the DISTAL atlas^68^ was used only for anatomical reference and visualization. In short, preoperative magnetic resonance imaging (MRI, MAGNETOM, 1.5 T Siemens) and postoperative computed tomography (CT, SOMATOM, Siemens) were used for electrode localization. Postoperative CT was acquired within 24 h after electrode implantation, in accordance with the clinical standard at our center. Images were co-registered and resampled to an isotropic resolution of 1 mm using B-spline interpolation. Postoperative electrode localization was carried out using the Lead-DBS toolbox version 2.6^67^. Postoperative CT or MRI images were co-registered to the preoperative MRI using either ANTs^69^ or SPM^70^, and then normalized to MNI ICBM 2009b NLIN ASYM space^71,72^ through ANTs-based nonlinear registration. A subcortical refinement step was applied to improve alignment in deep brain regions^73^. Electrode trajectories were then manually verified based on artifact visualization, and active contact coordinates were extracted in MNI space for subsequent analyses and predictions. Because visualization in common MNI space may give an inaccurate impression of the position of individual contacts relative to atlas borders, contacts were additionally checked in patient-native space, where needed, to confirm that they were located within, or in close proximity to, the STN border. Three-dimensional visualizations of the active contacts, STN anatomy, and SHAP-based feature attributions were created in Python 3.12^74^ using PyVista for surface rendering and color mapping^75^.

## Data Availability

Data are available upon request from the first author.

## Code availability

Code is available upon request from the first author.
